# Silver *N*-Heterocyclic Carbene (NHC) Complexes as Antimicrobial and/or Anticancer Agents

**DOI:** 10.3390/ph18010009

**Published:** 2024-12-25

**Authors:** Jessica Ceramella, Alessia Catalano, Annaluisa Mariconda, Assunta D’Amato, Saveria Aquila, Carmela Saturnino, Camillo Rosano, Maria Stefania Sinicropi, Pasquale Longo

**Affiliations:** 1 Department of Pharmacy, Health and Nutritional Sciences, University of Calabria, via Pietro Bucci, 87036 Arcavacata di Rende, Italy; jessica.ceramella@unical.it (J.C.); saveria.aquila@unical.it (S.A.); 2 Department of Pharmacy-Drug Sciences, University of Bari “Aldo Moro”, Via Orabona, 4, 70126 Bari, Italy; 3 Department of Basic and Applied Sciences, University of Basilicata, Via dell’Ateneo Lucano, 10, 85100 Potenza, Italy; annaluisa.mariconda@unibas.it; 4 Department of Chemistry and Biology “A. Zambelli”, University of Salerno, Via Giovanni Paolo II, 132, 84084 Fisciano, Italy; asdamato@unisa.it (A.D.); plongo@unisa.it (P.L.); 5 Department of Health Sciences, University of Basilicata, Via dell’Ateneo Lucano, 10, 85100 Potenza, Italy; carmela.saturnino@unibas.it; 6 U.O. Proteomica e Spettrometria di Massa, IRCCS Ospedale Policlinico San Martino, Largo Rosanna Benzi, 10, 16132 Genova, Italy; camillo.rosano@hsanmartino.it

**Keywords:** SBC3, silver–NHC, Ag(I)-NHC, precious metals, late-transition metals, catalysis, ESKAPE, mono-metal complexes

## Abstract

The strict connections/interactions between microbial infections and cancer are nowadays widely accepted. Hence, the dual (or multiple) targeting of microbial infections and cancer is an essential issue to be addressed. In this context, metal complexes have gained considerable importance and effectiveness in medicinal chemistry. Particularly, *N*-heterocyclic carbene (NHC) complexes with transition metals have emerged as very promising compounds. Among the myriad of NHC–metal complexes, those bearing silver will be the subject of this review. Numerous Ag(I)-NHC complexes have revealed high antibacterial and/or anticancer properties, even higher than those of reference drugs. Herein, we summarize the most recent studies while also discussing the proposed mechanism of action and offering an interesting remark about the research in this field. Literature databases (PubMed/MEDLINE, Scopus, and Google Scholar) were used as sources to search the literature, referring to the last five years.

## 1. Introduction

In recent years, the number of people suffering from multi-resistant microbial infections and cancer has arisen rapidly. The World Health Organization (WHO), as well as US and European governments, have identified antibiotic resistance and cancer as priority issues [1,2,3], considering the increasing cases of people affected by these pathologies [4,5]. ESKAPE pathogens (*Enterococcus faecium*, *Staphylococcus aureus*, *Klebsiella pneumoniae*, *Acinetobacter baumanii*, *Pseudomonas aeruginosa*, and *Enterobacter* species) and multidrug-resistant superbugs have been recognized as some of the most dangerous threats to human health [6,7]. The interconnection between these two health issues has, to date, been undoubtedly ascertained, as microbial infections are the second leading cause of comorbidity and death in patients with cancer [8]. In this view, the dual targeting of microbial infections and cancer is a crucial issue to address. Several studies dealing with this topic have been reported, including metal complexes [9,10,11], antimicrobial peptides [12,13,14], photodynamic therapy [15], amino acid derivatives [16], and nanomaterials [17]. Particularly, metal complexes, in which a metal is coordinated to different ligands, have gained considerable importance in medicinal chemistry for their different biological activities, as well as for their important role in catalysis and organic synthesis. Amongst them, *N*-heterocyclic carbene (NHC)–transition metal complexes have emerged as very promising metallodrugs [18,19,20]. Several NHC complexes with late-transition metals, such as silver (Ag), gold (Au), palladium (Pd), and ruthenium (Ru), have demonstrated interesting biological activities [21], including antibacterial [21,22,23,24,25], anticancer [26,27,28,29,30,31,32,33], or both [34,35,36,37,38]. Among the myriad of reported NHCs–metal complexes, the silver-based ones will be reviewed, given the great antimicrobial and antibiofilm properties [39,40,41] exerted via silver, the interesting results obtained for these compounds as antitumor, antioxidant, and antiparasitic agents [42,43,44,45,46,47,48], and, moreover, the wide use of these compounds as catalysts in organic and inorganic chemistry [49,50,51,52,53,54,55,56]. Several preclinical studies have also been conducted on silver–NHC complexes, but to our knowledge, none of them have reached phase 1 clinical studies so far [57]. NHC complexes bearing silver are generally Ag(I) complexes, although Pueyo et al. (2024) [58] recently reported the synthesis of Ag(III)–NHC complexes, exhibiting remarkable stability. Specifically, Ag(I)–NHC complexes are analyzed in this review, focusing particularly on the in vitro and in vivo studies carried out in the last five years regarding the antimicrobial or anticancer activities or both. A literature search was conducted on the PubMed/MEDLINE, Scopus, and Google Scholar search engines using general keywords such as “silver NHC”, “*N*-heterocyclic carbene”, “NHC complexes”, and “Ag-NHC”. All abstracts and full-text articles were examined for their relevance to this review.

## 2. Ag–NHC Complexes as Antimicrobials

Silver(I) has a long history of activity as an antimicrobial, and it has received increasing interest in the last decades, owing to the rise of antimicrobial resistance. The mechanism of antimicrobial activity has been widely studied. Positively charged silver (Ag+) ions bind to negatively charged cell membranes and induce cell membrane/wall leakage and/or rupture [59]. However, the main disadvantage is the limited duration of action of silver-containing antimicrobial agents. The bioavailability and activity of silver ions, Ag^+^, are influenced by the method of delivery and several environmental factors. For instance, the presence of certain anions, such as sulfides, phosphates, and chlorides, as well as specific cations like calcium and magnesium, can enhance Ag(I) bioactivity. Additionally, high temperatures and basic pH conditions also contribute to an increase in activity. Interestingly, due to their stability, NHC–silver complexes can release the active Ag^+^ cations over a prolonged time; this may represent a winning strategy for improving the antimicrobial activity [43,60,61,62,63]. The number of publications concerning the study of silver-based NHC complexes has increased considerably over the years, despite the antimicrobial mechanism not having been fully comprehended yet. Ronga et al. (2023) [60] recently summarized several studies regarding silver(I)–NHC complexes, also highlighting the mechanisms of antimicrobial activity. The authors found that, in order to show antimicrobial activity, complexes must meet two requirements; that is, they should retain their ligands releasing silver cation over a prolonged time, and they should guarantee sufficient lipophilicity, which allows the penetration of the complex into and across the membrane. Inside the cell, they deactivate the active enzyme sites of the microorganisms. The structure–activity relationships are related to the substituents on imidazole or benzimidazole rings at all positions. The antibacterial studies described below were carried out using the microdilution method, reporting the minimum inhibitory concentration (MIC) or the maximum growth inhibition (MGI), or via the well-pour method, using nutrient agar culture for bacterial growth, and the inhibitory zone diameter (IZD) is indicated. Beato et al. (2022) [64] used the Kirby–Bauer disk diffusion method and measured the zone of clearance (ZC).

### 2.1. SBC3 as an Antimicrobial Agent

**Figure 1:**
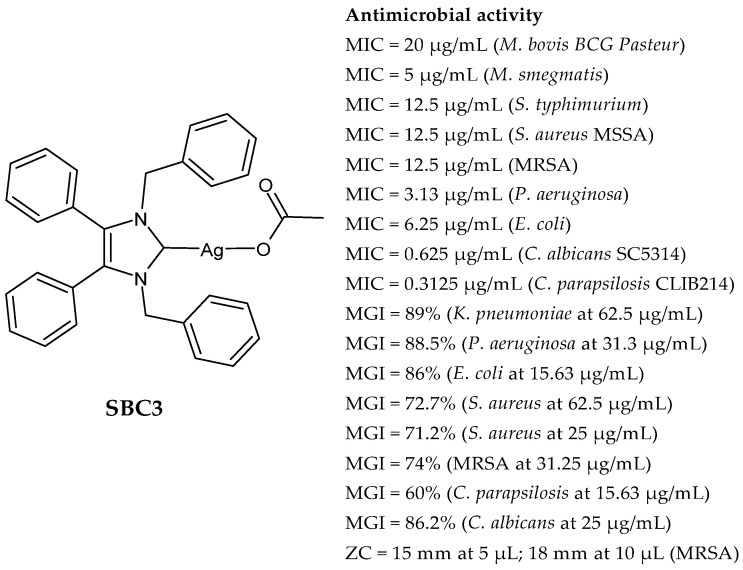
Structure of **SBC3** and its antimicrobial activity [64,65,66,68,70,72].

At the beginning of the last decade, the Ag–NHC acetate complex **SBC3** (Figure 1), which is specifically an *N*-heterocyclic carbene 1,3-dibenzyl-4,5-diphenylimidazol-2-ylidene silver(I) acetate, was reported [65]. MIC values against Gram-positive and Gram-negative bacteria (*Mycobacterium bovis BCG Pasteur*, *Mycobacterium smegmatis*, *Salmonella typhimurium*, *S. aureus* BH1CC, a methicillin-resistant *S. aureus* strain, BH1CC ΔSCC*mec*, an isogenic methicillin-sensitive derivative of BH1CC, *P. aeruginosa*, and *E. coli* (NCIB strain 9485)) are reported in the figure. Since then, several derivatives of SBC3, considered a lead compound, have been studied. **SBC3** demonstrated antibacterial activity against Gram-positive and Gram-negative bacteria and antifungal activity against *Candida albicans* and *Candida parapsilosis*. An interesting antibacterial action was also demonstrated against multidrug-resistant *S. aureus* (Gram-positive) and *P. aeruginosa* (Gram-negative), which are associated with chronic wound infections and cystic fibrosis lung colonization, respectively. The in vivo activity of **SBC3** against *S. aureus* and *C. albicans* was demonstrated for the first time by Browne et al. in 2014 in *Galleria mellonella* larvae at three different times (24, 48, and 72 h) [66]. In 2019 and 2021, the research group of O’Beirne [67,68] reported two studies on **SBC3** and analyzed its antibacterial mechanism of action as an inhibitor of bacterial thioredoxin reductase, also behaving as an antibacterial adjuvant for gentamicin against resistant strains of *P. aeruginosa*. Then, the proteomic response of *S. aureus*, *P. aeruginosa*, and *C. parapsilosis* to **SBC3** was examined by the same research group in 2022 and 2023 [69,70] using the label-free proteomics technique. In *C. parapsilosis*, **SBC3** reduced adherence to epithelial cells and biofilm formation, thus reducing fungal virulence. In *C. parapsilosis*, the authors found a considerable increase in the abundance of chitinase that may also be responsible for the antifungal activity, as chitin is a polysaccharide component of the fungal cell wall that provides structural stability to the cell. Regarding the antibacterial mechanism of action, **SBC3** showed different activities in *P. aeruginosa,* and *S. aureus*. Specifically, in *P. aeruginosa* it enhanced the alginate biosynthesis, the secretion system, and drug detoxification proteins, and it reduced anaerobic respiration, twitching motility, and ABC transport. Oppositely, in *S. aureus*, it enhanced DNA replication/repair and cell redox homeostasis and reduced protein synthesis, lipoylation, and glucose metabolism. However, the structural damage induced via **SBC3,** caused by the increase in the abundance of cell wall/membrane proteins, was demonstrated in both bacteria. Recently, Chen et al. (2022) [71] demonstrated a synergistic effect of **SBC3** with ebselen, a seleno-organic drug targeting bacterial thioredoxin reductase (bTrxR) against *E. coli* infection, based on the direct inhibition of *E. coli* TrxR and the depletion of glutathione (GSH), resulting in the up-regulation of the reactive oxygen species (ROS) level and causing the bacteria death. The bactericidal effect of this combination was also confirmed in vivo in mild and acute *E. coli* BC1-induced peritonitis in mice. In addition, the combination of **SBC3** with ebselen enhanced the antibacterial activity by up to 80 times against Gram-negative bacteria (*E. coli*, *A. baumannii*, *E. cloacae*, *K. pneumoniae*, and *P. aeruginosa*).

### 2.2. Other Ag–NHC Complexes as Antimicrobial Agents

Studies regarding Ag(I)–NHC derivatives of **SBC3** and other Ag(I)–NHCs acting as antimicrobials are summarized in Table 1. In 2021, O’Beirne et al. [72] reported the study of some derivatives of **SBC3** as antimicrobials against Gram-positive bacteria (*S. aureus* and methicillin-resistant *S. aureus*, MRSA), Gram-negative bacteria (*E. coli*, *K. pneumoniae*, and *P. aeruginosa*), and fungi (*C. albicans* and *C. parapsilosis*). The compounds were obtained in high yields via a continuous flow synthetic process in two different ways by using stoichiometric amounts of silver oxide or excess silver oxide in a 3:1 solvent mixture of dry toluene/methanol. The latter method provided final compounds in higher yields (86%, 96%, and 97% for **1**, **2**, and **3**, respectively). The in vitro evaluation of MGI demonstrated good activity for compounds **1**–**3,** as well as for the lead compound **SBC3**. Moreover, the ability to inhibit the biofilm formation in MRSA and *C. parapsilosis* was demonstrated. Specifically, complexes **SBC3** and **1** showed interesting activity against both MRSA and *C. parapsilosis*, whereas **2** was more active against *C. parapsilosis* and complex **3** against MRSA. Complexes **SBC3** and **1** were also tested in vivo, using a murine model against infected with a strain of MRSA (ATCC 33591). The promising inhibition of MRSA was demonstrated using complex **SBC3**; however, at concentrations higher than 20 mg/kg, toxicity was observed for both **SBC3** and complex **1,** with all mice in these test sub-groups dying during the treatment.

In 2022, Beato et al. [64] studied two series of fluoro-substituted cationic and neutral NHC silver derivatives of **SBC3** as antibacterials, using the Kirby–Bauer test. New complexes were tested in triplicate, and **SBC3** and tetracycline were tested once. The fluorine derivative of **SBC3**, namely complex **4,** showed the same activity of **SBC3** against MRSA after 24 h of incubation (tetracycline: ZC = 24 mm and 26 mm at 5 µL and 10 µL, respectively). Complex **4** was prepared with a 65% yield via the reaction of the corresponding imidazolium bromide salt with two equivalents of silver acetate in dichloromethane.

Muniyappan et al. (2021) [73] studied some picolyl- and benzyl-linked biphenyl NHC ligands and their silver(I)–NHC complexes as antimicrobials against *S. aureus* IE903, *S. aureus* E321, and *P. aeruginosa* E322 clinical isolates obtained from urine/pus from three different patients, after 48 h of incubation. The complexes were synthesized via the reaction of NHC ligands and silver (I) oxide at room temperature in the absence of light, followed by the counter anion exchange with potassium hexafluorophosphate. For the antimicrobial evaluation, the microbroth dilution method was used, and since this was a preliminary pilot study, a comparison with a standard antimicrobial agent was not performed. Complexes **5** and **6** were synthesized in good yields (81% and 74%, respectively), and showed medium to high activity against only one of the two strains of *S. aureus* (IE903) and *P. aeruginosa*. In particular, complex **5** showed the same activity of the ligand against *S. aureus* (IE903), while it was more active than the ligand against *P. aeruginosa*. Complex **6** was 4-fold and 10-fold more active than the ligands against *S. aureus* (IE903) and *P. aeruginosa*, respectively.

Prencipe et al. (2021) [74] studied two series of Ag–NHC complexes based on an acridine scaffold, specifically mono NHC–Ag neutral and bis NHC–Ag cationic complexes, as antimicrobials against two Gram-negative (*E. coli* DH5 and *P. aeruginosa* PAOI) and two Gram-positive (*S. aureus* 6538P and *B. subtilis* PY79) bacteria (overnight incubation). Each experiment was performed at least in triplicate, and standard deviations were less than 5%. The most interesting compounds were the dinuclear complexes, **7** and **8**, and the mononuclear complex **9**, the synthesis of which had been previously described by the same research group [75], showing MIC values below 1 µM. For comparison, the authors referred to Patil et al. (2015) [76] and assessed that this class of complexes was active at a concentration 100 times lower than the NHC–silver complexes previously tested. The new complexes were always more active than the corresponding ligands.

Nadeem et al. (2022) [77] reported the study of silver(I)–NHC complexes from bis-benzimidazolium salts (**10** and **11**) as antibacterials at a concentration of 10 mg/mL (in triplicate, 24 h of incubation) against Gram-positive *S. aureus* and Gram-negative *E. coli*, using ciprofloxacin (at 10 mg/mL concentration) as the standard drug (IZD = 34.67 ± 1.15 mm for *S. aureus* and 35 ± 1 mm for *E. coli*). The synthesis was obtained using Ag_2_O, KPF_6_ in MeOH, as described in the article obtaining **10** and **11** in 47% and 62% yields, respectively. Complexes **10** and **11** showed higher activity than ciprofloxacin against both bacteria and were also more active than free ligands.

Bensalah et al. (2023) [78] reported a series of Ag(I)–NHCs complexes (**12**–**16**) active against Gram-negative bacterial strains (*E. coli*, *P. aeruginosa*, and *K. pneumoniae*) and Gram-positive bacterial strains (*S. aureus* and MRSA). Complexes were prepared using an in situ deprotonation method with Ag_2_O as the main silver, obtaining final complexes as white solids in good yields (78%, 34%, 80%, 58%, and 62% for **12**, **13**, **14**, **15**, and **16**, respectively). Complex **14** was the most active against *E. coli* (standard ampicillin: MIC = 18 ± 0.5 µmol/L), complex **16** against *P. aeruginosa* (no standard used for comparison), complex **13** against *K. pneumoniae* (standard ampicillin: MIC = 3 ± 0.9 µmol/L), complexes **14** and **15** against *S. aureus* (standard ampicillin: MIC = 10 ± 0.2 µmol/L), and complex **12** against MRSA (no standard used for comparison). Complex **12** was also active against *C. albicans* (standard fluconazole: MIC = 3.12 ± 0.2 µmol/L).

**Table 1:** Ag–NHC complexes as antimicrobials.

Compd		Antibacterial Activity	Ref
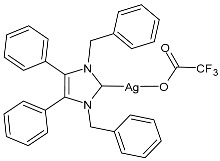	**1**	MGI = 85% (*K. pneumoniae* at 15.63 μg/mL) MGI not given (*P. aeruginosa)* MGI = 86% (*E. coli* at 7.8 μg/mL) MGI = 73% (*S. aureus* at 15.63 μg/mL) MGI = 79% (MRSA at 31.25 μg/mL) MGI = 77% (*C. parapsilosis* at 7.8 μg/mL) MGI = No inhibition (*C. albicans*)	[72]
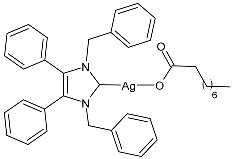	**2**	MGI = 87% (*K. pneumoniae* at 15.63 µg/mL) MGI = 84% (*P. aeruginosa* at 15.63 µg/mL) MGI = 86% (*E. coli* at 7.8 μg/mL) MGI = ∼ 50% (*S. aureus* at 15.63 μg/mL) MGI = ∼ 50% (MRSA at 15.63 μg/mL) MGI = 77% (*C. parapsilosis* at 15.63 μg/mL) MGI = 95% (*C. albicans* at 62.5 µg/mL)	[72]
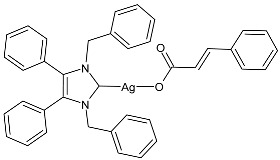	**3**	MGI not given (*K. pneumoniae)* MGI not given (*P. aeruginosa)* MGI = 82% (*E. coli* at 15.63 µg/mL) MGI = not given (*S. aureus*) MGI = 76% (MRSA at 62.5 µg/mL) MGI = not given (*C. parapsilosis*) MGI = 94% (*C. albicans* at 125 µg/mL)	[72]
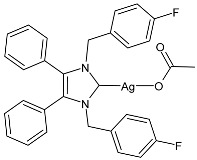	**4**	ZC = 15 mm at 5 µL; 18 mm at 10 µL (MRSA)	[64]
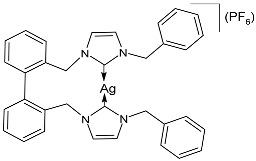	**5**	MIC = 50 mg/L (*S. aureus IE903*) MIC = 200 mg/L (*S. aureus E321*) MIC = 10 mg/L (*P. aeruginosa E322*)	[73]
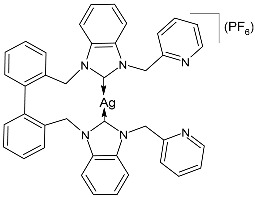	**6**	MIC = 50 mg/L (*S. aureus IE903*) MIC = 100 mg/L (*S. aureus E321*) MIC = 10 mg/L (*P. aeruginosa E322*)	[73]
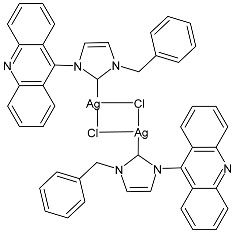	**7**	MIC ≤ 1 µM (*E. coli* DH5α) MIC ≤ 1 µM (*P. aeruginosa* PAOI) MIC ≤ 1 µM (*B. subtilis* PY79) MIC ≤ 1 µM (*S. aureus* 6538P)	[74]
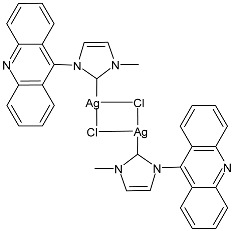	**8**	MIC ≤ 1 µM (*E. coli* DH5α) MIC ≤ 1 µM (*P. aeruginosa* PAOI) MIC ≤ 1 µM (*B. subtilis* PY79) MIC ≤ 1 µM (*S. aureus* 6538P)	[74]
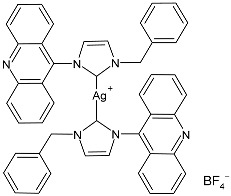	**9**	MIC ≤ 1 µM (*E. coli* DH5α) MIC ≤ 1 µM (*P. aeruginosa* PAOI) MIC ≤ 1 µM (*B. subtilis* PY79) MIC ≤ 1 µM (*S. aureus* 6538P)	[74]
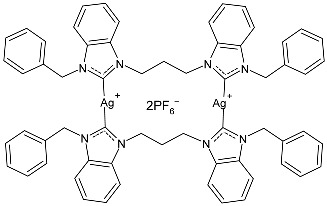	**10**	IZD = 18.67 ± 0.58 mm (*S. aureus*) IZD = 12.5 ± 0.5 mm (*E. coli*)	[77]
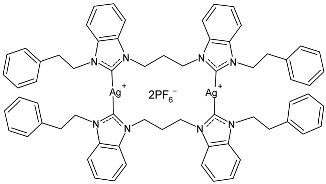	**11**	IZD = 19.83 ± 0.29 mm (*S. aureus*) IZD = 14.33 ± 0.58 mm (*E. coli*)	[77]
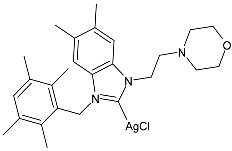	**12**	MIC = 26 ± 0.7 µmol/L (*E. coli* ATCC 25988) MIC = 80 ± 1.8 µmol/L (*P. aeruginosa* ATCC 27853) MIC = 22 ± 0.6 µmol/L (*K. pneumoniae* ATCC 700603) MIC = 15 ± 0.3 µmol/L (*S. aureus* ATCC 29213) MIC = 11 ± 0.2 µmol/L (MRSA ATCC 43300) MIC = 8 ± 0.6 µmol/L (*C. albicans* ATCC 14053)	[78]
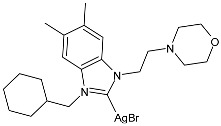	**13**	MIC = 26 ± 0.3 µmol/L (*E. coli* ATCC 25988) MIC = 51 ± 1.2 µmol/L (*P. aeruginosa* ATCC 27853) MIC = 14 ± 0.2 µmol/L (*K. pneumoniae* ATCC 700603) MIC = 14 ± 0.6 µmol/L (*S. aureus* ATCC 29213) MIC = 13 ± 0.4 µmol/L (MRSA ATCC 43300) MIC = 14 ± 0.3 µmol/L (*C. albicans* ATCC 14053)	[78]
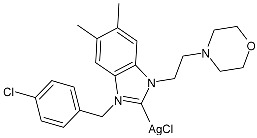	**14**	MIC = 23 ± 0.8 µmol/L (*E. coli* ATCC 25988) MIC = 26 ± 1.3 µmol/L (*P. aeruginosa* ATCC 27853) MIC = 24 ± 0.8 µmol/L (*K. pneumoniae*) ATCC 700603 MIC = 12 ± 0.4 µmol/L (*S. aureus* ATCC 29213) MIC = 27 ± 1.1 µmol/L (MRSA ATCC 43300) MIC = 15 ± 0.2 µmol/L (*C. albicans* ATCC 14053)	[78]
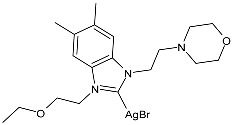	**15**	MIC = 26 ± 0.8 µmol/L (*E. coli* ATCC 25988) MIC = 27 ± 0.8 µmol/L (*P. aeruginosa* ATCC 27853) MIC = 26 ± 0.9 µmol/L (*K. pneumoniae* ATCC 700603) MIC = 12 ± 0.4 µmol/L (*S. aureus* ATCC 29213) MIC = 26 ± 0.7 µmol/L (MRSA ATCC 43300) MIC = 14 ± 0.2 µmol/L (*C. albicans* ATCC 14053)	[78]
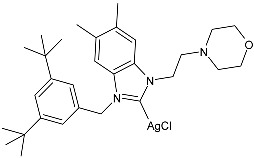	**16**	MIC = 27 ± 0.9 µmol/L (*E. coli* ATCC 25988) MIC = 24 ± 0.9 µmol/L (*P. aeruginosa* ATCC 27853) MIC = 24 ± 0.8 µmol/L (*K. pneumoniae* ATCC 700603) MIC = 17 ± 0.2 µmol/L (*S. aureus* ATCC 29213) MIC = 27 ± 0.5 µmol/L (MRSA ATCC 43300) MIC = 13 ± 0.2 µmol/L (*C. albicans* ATCC 14053)	[78]

MIC = minimum inhibitory concentration; ZC = zone of clearance; MGI = maximum growth inhibition; IZD = inhibitory zone diameter.

## 3. Ag–NHC Complexes as Anticancer Agents

The discovery of the anticancer potential of silver(I)–NHC complexes was an unexpected discovery that is still being explored. Cytotoxicity studies are summarized in Table 2, and the half-maximal (50%) inhibitory concentration (IC_50_) values are given. The MTT (3-(4,5-dimethylthiazol-2-yl)-2,5-diphenyltetrazolium bromide) cell viability test was adopted to evaluate the cytotoxic effects of the complexes, unless otherwise indicated. The standard used for comparison and cytotoxicity evaluation against non-tumoral cells, when given, is detailed in the text.

Guarra et al. (2020) [79] studied two complexes bearing an NHC ligand, specifically silver(I) and gold(I) complexes, further functionalized with an anthracenyl moiety (specifically, 1-(9-anthracenylmethyl)-3-(3-trimethylsilyl-2-propynil)-benzimidazol-2-ylidene) as anticancer agents by means of the MTT assay. In Table 2, only complex **17** is shown (whereas the corresponding contains Au–Cl instead of Ag–Br). It was prepared in a 56% yield via the reaction of the ligand with 0.7 equivalents of Ag_2_O in CH_2_Cl_2_. This compound showed cytotoxicity in the micromolar range and antiproliferative properties higher than the ligand and the reference cisplatin against SW480 (colon), A549 (lung), and HepG2 (liver) cell lines. IMR-90 (lung fibroblasts) were used as healthy cell lines. Auranofin (a gold antiarthritic and anti-rheumatic drug commonly used as a positive control for gold complexes) and cisplatin were used as references. The IC_50_ values for reference drugs (evaluated after 24 h) were as follows: auranofin IC_50_ = 0.7 ± 0.1 µM and 1.2 ± 0.1 µM against SW480 and HepG2, respectively; cisplatin IC_50_ = 47 ± 1 µM, 38 ± 2 µM and 29 ± 1 µM against SW480, A549 and HepG2, respectively. Regarding their selectivity towards cancer cells, they can be considered selective as cisplatin. The TrxR (IC_50_) values were reported as the means ± SDs of three experiments. Both complexes were internalized by SW480 cells, and the Ag(I) complex was the most accumulated. They were analyzed as inhibitors of the selenoenzyme thioredoxin reductase (TrxR) and DNA binders. Fluorescence microscopy confirmed that both TrxR and DNA binding could be involved in the biological activity of the complexes. The complex with silver (**17**) was the most potent TrxR inhibitor, with an IC_50_ in the nanomolar range. The authors suggest that the metal center tunes the biological features, as Ag(I)–NHC intercalates into DNA, whereas Au(I)–NHC does not. Also, interaction studies with natural double-stranded DNA highlighted a strong stabilization of the double helix after binding to Ag(I)–NHC, suggesting its potential suitability as dual-targeting anticancer agent.

Çevik-Yildiz et al. (2020) [80] studied the Ag(I)–NHC complexes **18**–**20** as anticancer agents against human breast (MCF-7, MDA-MB-231) and prostate (DU-145) cancer cell lines by means of an MTT assay after 72 h. Data were based on the means from at least three independent experiments, each comprising three replicates per concentration. The complexes were prepared via a reaction with AgNO_3_ in methanol in 81%, 84%, and 87% yields for **18**, **19**, and **20**, respectively. Complexes **18**–**20** showed dose- and time-dependent cytotoxic activity against all cell lines and were generally more active than the corresponding ligands. MDA-MB-231 and MCF-7 human breast carcinoma cells were the most sensitive to the complexes. IC_50_ values in normal cells (L-929) were higher, suggesting the selectivity of the complexes for human breast cancer cells. Complex **18** showed high selectivity for MDA-MB-231 cancer cells, whereas complex **19** showed selectivity against MCF-7 breast cancer cells.

Atif et al. (2020) [81] described the anticancer studies of three silver–NHC complexes, symmetrically and non-symmetrically substituted (**21**–**23**). They were prepared by treating the corresponding azolium salts with silver oxide in methanol, obtaining complexes **21**, **22**, and **23** in 56%, 58%, and 61% yields, respectively. Complex **21** was characterized by the single-crystal X-ray diffraction technique, showing a linear C–Ag–C coordination geometry for the silver(I) center. In vitro studies were carried out against human colorectal cancer (HCT-116), breast cancer (MCF-7), and erythromyeloblastoid leukemia (K-562) cell lines via an MTT assay for 72 h, whereas in vivo acute oral toxicity (IAOT) was determined through the evaluation of agility and body weight in female rats. Reference compounds were 5-fluorouracil (IC_50_ = 5.2 µM against HCT-116), tamoxifen (IC_50_ = 5.5 µM against MCF-7), and betulinic acid (IC_50_ = 17.0 µM against K-562). The complexes were more active than the ligands. The most interesting ones were **21** and **23**, but the complex **21** was 10- and 2-fold more active than standard drugs against HCT-116 and K-562 cell lines, respectively. The increased antiproliferative effect of silver complexes was associated with cellular apoptosis and cell-cycle arrest. In vivo studies demonstrated vigor and agility in all the tested animals, which suggests the biocompatibility and non-toxicity of the compounds.

Md Zin et al. (2022) [82] studied a series of asymmetrical *N*,*N’*-disubstituted benzimidazolium NHC ligands and their mononuclear Ag(I) complexes (**24**–**27**) as anticancer agents against human cervical cancer cells (HeLa) by means of the MTT assay using etoposide as is standard [IC_50_ = 15.11 ± 1.23 µg/mL (25.67 μM)]. IC_50_ values were determined after 24 h. Complexes **24**–**27** were more active than etoposide, but they also showed activity against normal human skin fibroblasts (Hs27). Data demonstrated that the incorporation of the silver(I) ions into the ligands greatly enhanced their cytotoxic effect. Complexes were synthesized by stirring a mixture of the corresponding ligand and Ag_2_O in methanol in moderate yields (56%, 49%, 46%, and 47% for **24**, **25**, **26**, and **27**, respectively).

Hobsteter et al. (2024) [83] prepared and characterized four stable acetylated D-galactopyranoside-incorporated Ag–NHC complexes (**28**–**31**), obtained with an anomeric β-configuration and as monocarbene species. The imidazolium salts and silver oxide were added to dry CH_2_Cl_2_ under a nitrogen atmosphere, and complexes **28**, **29**, **30**, and **31** were obtained in high yields (95%, 96%, 87%, and 90%, respectively). They were studied for their anti-proliferative activity in rhabdomyosarcoma (RMS), a highly aggressive form of cancer that develops from mesenchymal cells that have failed to fully differentiate into myocytes of skeletal muscle. The crystal violet staining technique was used to determine cell growth, and IC_50_ values were obtained against the human RMS cell line after 72 h. The complexes demonstrated concentration-dependent anti-growth effects and complex-specific activation patterns in the Akt 1/2, ERK 1/2, and p38-MAPK signaling pathways.

Ashraf et al. (2024) [84] studied two binuclear silver–NHC complexes (**32** and **33**) as anticancer agents against HCT-116, a lung cancer cell line (A549), and a breast cancer cell line (MCF-7) through an MTT assay using drug 5-flourouracil (5-FU), oxaliplatin, and cisplatin as standard drugs. The calculated IC_50_ values were calculated after 48 h of incubation. The values of standards were the following: cisplatin (against A549 and A-2780): IC_50_ = 0.813 ± 0.06 µg/mL and 0.786 ± 0.03 µg/mL, respectively; oxalilplatin (against A549 and A-2780): IC_50_ = 0.809 ± 0.05 µg/mL and 0.764 ± 0.06 µg/mL, respectively; 5-fluorouracil (against MCF-7 and HCT-116): IC_50_ = 0.998 ± 0.06 µg/mL and 0.996 ± 0.04 µg/mL, respectively. The in vitro assays evidenced that the complexes showed higher cytotoxicity than the ligands, with complex **33** being the most active, especially against MCF-7 and HCT-116 cell lines, with a higher cytotoxic effect than the reference 5-fluorouracil but slightly lower than cisplatin and oxalilplatin. The synthesis of the complexes was obtained by stirring a mixture of benzimidazolium salt and Ag_2_O in methanol. After filtration through celite, it was further reacted for metathesis with an aqueous solution of KPF_6_. Stability in solutions was demonstrated for all complexes at different times. Aqueous solubility was likely improved via the interactions of **32** and **33** with surfactants SDS, tween-20, and tween-80. The strongest binding affinity was found for SDS (sodium dodecyl sulfate) with respect to tween-20 and tween-80. The molecular modeling study of complex **32** suggested a potential interaction with the aromatase cytochrome P450, which could be related to anticancer activity.

The group of Hamdi and collaborators recently reported two papers [85,86] regarding two series of silver–NHC complexes bearing a benzimidazole moiety and evaluated their anticancer activity against the HepG2, A549, and MCF-7 cancer cell lines, using cisplatin as a reference. In ref. [85], the IC_50_ values for cisplatin were as follows: 16.46 ± 4.75 µM; 18.5 ± 3.25 µM; and 4.14 ± 2.12 µM, respectively. IC_50_ values were performed in duplicate. The complexes were generally more active than the ligands. Complexes **34**–**36** were the most active of the first series [85] against HepG2 and A549, being more active than the reference drug against one or both cell lines. Complexes **37**–**39** of the second series [86] were the more active of those belonging to the first one. The synthesis was obtained via the in situ deprotonation of benzimidazolium salts method using Ag_2_O in moderate yields (67%, 68%, 77%, 80%, 80%, and 88% for **34**, **35**, **36**, **37**, **38**, and **39**, respectively).

## 4. Ag–NHC as Dual Anticancer and Antibacterial Agents

The most intriguing studies have addressed complexes with dual activity, acting as both antimicrobial and anticancer agents. They are summarized in Table 3.

Sharhan et al. (2020) [87] reported the study of series of *N*-alkylated benzimidazolium salts based on 9-substituted acridine and the corresponding Ag(I) carbene complexes as anticancer agents against MCF-7 breast cancer cell lines (the incubation period was 24 h) and antibacterial drugs against Gram-positive (*S. aureus* ATCC 25923 and a clinical isolate of *Staphylococcus epidermidis*) and Gram-negative (*P. aeruginosa* ATCC27853 and clinical isolates of *E. coli* and *Salmonella typhi*) bacteria (24 h incubation). Tamoxifene and paclitaxel were used as standard drugs for anticancer activity evaluation [IC_50_ = 11 ± 1 mg/mL (30 µM) and 6 ± 1 mg/mL (7 µM), respectively], whereas ampicillin (at 10 µg/mL concentration) was used as the standard for antimicrobial evaluation (MIC = 125 mg/mL against Gram-positive bacteria and MIC = 250, 125 and 250 mg/mL against *P. aeruginosa*, *E. coli,* and *S. typhi*, respectively). All activity studies were performed in triplicate. The complexes showed generally higher activity than the ligands as both anticancer and antimicrobials. Complexes **40** and **41** were the most interesting of the series, even though their activity was lower than that of standard drugs. The complexes demonstrated no significant cytotoxic effects towards the non-tumorigenic breast cell line MCF-10A with IC_50_ values above 50 μg/mL. For the synthesis, the ligand precursors were reacted with Ag_2_O to provide carbene complexes in moderate yields (63% and 64%, respectively).

In the paper by Sirignano et al. (2022) [53], the synthesis of Ag(I) and Au(I) complexes stabilized via NHC with hydroxy derivatives on nitrogen atoms and the biological activity evaluation as antibacterial (against both Gram-positive and Gram-negative bacteria) and anticancer agents (against breast cancer cell lines) were reported. Antibacterial studies revealed that the Ag–NHC complexes were selective for *E. coli*, while the NHC–Au analogs showed activity against *S. aureus*. For each compound, the MIC was repeated in three independent experiments, each in triplicate (24 h incubation). For cytotoxicity studies, all the calculations were performed in triplicate. The IC_50_ values represent the mean ± standard deviation (n = 3). Additionally, the *N*,*N’*-hydroxy derivatives of Ag–NHC complexes **42** and **43** demonstrated significant anticancer activity against MCF-7 and HeLa cancer cell lines, with higher activity than cisplatin, used as a reference (IC_50_ = 28.9 ± 0.7 µM, 36.2 ± 1.0 µM, 16.2 ± 1.1 µM, against MDA-MB-231, MCF-7 and HeLa, respectively). The cells were exposed to the target compounds dissolved in DMSO for 72 h. The complexes were not cytotoxic against normal breast MCF-10A or embryonic kidney Hek-293 cell lines. The synthesis was obtained by suspending the imidazolium salts in dry dichloromethane; then, Ag_2_O was added to the mixture, achieving the desired compounds in 50% and 55% yield, respectively.

Jakob et al. (2021) [88] described the synthesis and biological evaluation of three macrocyclic Cu(I), Ag(I), and Au(I) tricarbene/urea NHC complexes as antibacterial agents against *S. aureus* and *E. coli* and anticancer agents against MCF-7 breast and HeLa cervix carcinoma cell lines. IC_50_ and MIC values were determined after 24 h and 18 h, respectively. All experiments were performed in technical triplicates. The most interesting results were obtained for the complex with silver (**44**), obtained at a 44% yield via the reaction of the corresponding tricationic macrocyclic imidazolium salt with 3.0 equivalents of Ag_2_O in MeCN. AgNO_3_ and cisplatin were used as standards. Complex **44** showed interesting antiproliferative activity and moderate antimicrobial activity. MIC = 33 µM and 37 µM against *S. aureus* and *E. coli*, respectively, for AgNO_3_; IC_50_ = 39.9 ± 4.6 µM and 18.1 ± 5.1 µM against HeLa and MCF-7, respectively. It is interesting to note that the corresponding copper complex showed moderate antiproliferative activity, while it was inactive against bacteria; the corresponding gold complex was inactive as either an antiproliferative or an antibacterial.

In 2022, Mariconda et al. [89] reported a study on the antibacterial and anticancer activities of some NHC–silver complexes. Regarding MIC evaluation, for each experiment, carried out five times, triplicate assays were performed. All the experiments for cytotoxicity evaluation were performed in triplicate. Complexes were prepared via salt metathesis reactions between the corresponding bis asymmetric NHC–silver(I) iodide complexes and silver acetate in dichloromethane or methanol, obtaining yields in the range of 59–71%. The most interesting compounds were **45**–**47**, which showed antibacterial activity after 24 h of incubation against the Gram-positive bacteria *S. aureus* and *E. faecalis* and the Gram-negative bacterium *E. coli.* All strains used were ampicillin-sensible. The anticancer activity was evaluated against two breast cancer cell lines, namely MDA-MB-231 and MCF-7, compared to standard latrunculin A (IC_50_ = 2.45 ^×^ 10^−2^ ± 0.9 µM against MDA-MB-231 and IC_50_ = 0.14 ± 1.0 µM against MCF-7) and cisplatin (IC_50_ = 28.7 ± 0.4 µM against MDA-MB-231 and IC_50_ = 35.8 ± 0.7 µM against MCF-7). IC_50_ values were determined after 72 h. The most active complexes were **45** and **47**. Specifically, complex **45** was more active on the triple-negative MDA-MB-231 cells, whereas **47** displayed higher anticancer activity on the MCF-7 cells. All the tested complexes did not show any cytotoxic effect on MCF-10A normal cells. The complexes inhibited in vitro the activity of the human topoisomerases I and II and interfered with the cytoskeleton dynamic, as was also confirmed via in silico studies.

Sarfraz et al. (2022) [90] studied four silver–NHC complexes and their anticancer, antibacterial, and antioxidant activities. The most interesting complexes were **48**–**50**. Cytotoxicity studies were carried out through an MTT assay against the MCF-7, HCT-116, and A549 cell lines, and approved drugs (5-flourouracil, carboplatin, and cisplatin) were used as references (5-flourouracil: IC_50_ = 0.9978 ± 0.06 µg/mL and 0.9981 ± 0.11 against MCF-7 and HCT-116, respectively; carboplatin: IC_50_ = 0.803 µg/mL against A549; cisplatin: IC_50_ = 0.814 µg/mL against A549). IC_50_ values were determined after 48 h and measured in triplicates. Compound **49** was the most active as anticancer agent, with even higher activity than 5-fluorouracil against MCF-7 cancer cell lines. Antibacterial activity against bacterial strains (*S. aureus*, *M. luteus*, *E. coli,* and *S. typhimurium*; ATCC was not reported) was obtained through the well diffusion method and MIC through the microtiter plate method. All the experiments were conducted in triplicate. Ciprofloxacin was used as is standard (MIC = 0.0121 ± 0.0003 µg/mL, 0.015 ± 0.0002 µg/mL, 0.021 ± 0.004 µg/mL; 0.031 ± 0.002 µg/mL against *S. aureus*, *M. luteus*, *E. coli*, and *S. typhimurium*, respectively). The most active complex was **50,** followed by **49**.

Ulu et al. (2023) [91] reported a very interesting study synthesizing promising antibacterial and anticancer agents (**51**–**54**). Ag(I)–NHC complexes were obtained from the reaction of benzimidazolium salts with Ag_2_O in dichloromethane using Schlenk techniques (yields: 71%, 61%, 73%, and 74% for **51**, **52**, **53** and **54**, respectively) and studied in vitro as anticancer agents against a breast adenocarcinoma cell line (MCF-7) using an MTT assay and as antibacterial agents (10 μL of the compounds at 1 mg/mL concentration were used) against *B. subtilis* as Gram-positive bacterial strains and *E. coli* as Gram-negative bacterial strains, using ampicillin as a standard antibiotic at the same concentration. Experiments were performed at least in triplicate. All complexes showed good activity against bacteria, with complexes **52** and **53** showing higher bactericidal activity against *B. subtilis* than ampicillin (IZD = 6.67 ± 1.15 mm and 39.33 ± 5.03 mm against *B. subtilis* and *E. coli*, respectively). The activity of **53** was about 1.5-fold higher than that of ampicillin against *B. subtilis*. All experiments were performed at least in triplicate. In the cytotoxicity studies, all complexes were more active than cisplatin (IC_50_ = 82.02 ± 6.19 µg/mL) after 24 h incubation; in particular, complexes **51** and **53** demonstrated the highest anticancer activity, with **53** being about 24 times more active than cisplatin. However, the complexes should also be tested against non-tumorigenic cell lines.

Mariconda et al. (2024) [92] reported a study on silver and gold complexes with NHC derived from caffeine and their biological activities as anticancers and antibacterials. Complex **55** was synthesized following the synthetic procedure published by Phillips and Willams et al. through the reaction of the corresponding caffeine-derived salt with 2 equivalents of silver acetate in acetonitrile (yield 40%), showing high anticancer activity, which was even more active than cisplatin against MDA-MB-231 cells (cisplatin, IC_50_ = 32.2 ± 1 µM against MDA-MB-231; IC_50_ = 26.2 ± 1 µM against MCF-7). IC_50_ values were determined after 72 h as the means ± standard deviations of three different experiments performed in triplicate. The complexes were also tested against Gram-positive (*S. aureus*, *E. faecalis*, and *S. epidermidis*) and Gram-negative (*E. coli*, *K. pneumoniae*, *P. aeruginosa,* and *S. typhimurium*) bacteria. Complex **55** showed slight activity against *S. aureus*, *E. coli*, *S. epidermidis*, and *P. aeruginosa* (after 24 h of incubation), which were sensitive to ampicillin. The results were representative of three independent experiments performed in triplicate. The authors found a reduction in TNF-α expression of about 50% that may be further investigated, since TNF-α can act as an endogenous cancer promoter, bridging inflammation and carcinogenesis, and it has been found to be highly expressed in different preneoplastic and tumor tissues.

Karci et al. (2024) [93] reported the synthesis of a series of Ag–NHC complexes containing the benzimidazole moiety and their biological evaluation as antimicrobial and anticancer agents. Specifically, antifungal activity was studied against *C. albicans* and *Candida glabrata*, whereas antibacterial activity was run on *E. coli*, *P. aeruginosa*, and *S. aureus*. Anticancer studies were accomplished against A549, MCF-7, HCT116, and SH-SY5Y (neuroblastoma) cancer cell lines. Anticancer activity measurements were carried through an alamarBlue assay. IC_50_ values were determined after 24 h in triplicate. Complex **56** was prepared with the method of Organ through the reaction of benzimidazolium salts with Ag_2_O in dry chloroform (yield 34%). It showed high activity as both an antimicrobial and anticancer agent. The references used for antimicrobial activities were amphotericin B and voriconazole against fungi (amphotericin B, MIC = 0.05 and 0.1 µg/mL against *C. albicans* and *C. glabrata*, respectively; voriconazole, MIC = 0.4 µg/mL against *C. albicans* and *C. glabrata*) and ampicillin and tetracycline against bacteria (ampicillin, MIC = 12.5, 400 and 3.25 µg/mL against *E. coli*, *P. aeruginosa,* and *S. aureus*, respectively; tetracycline, MIC = 0.8, 12.5 and 0.2 µg/mL against *E. coli*, *P. aeruginosa,* and *S. aureus*, respectively): the reference for anticancer activity was cisplatin (IC_50_ = 205.01 ± 4.45, 76.31 ± 3.62, 310.12 ± 3.09 and 152.12 ± 4.65 µM, against A549, MCF-7, HCT-116, and SH-SY5Y, respectively). BEAS-2B (bronchial epithelial cells) were used as healthy cells for comparison, showing low residual toxicity for the complexes.

## 5. Conclusions

One of the most important medical discoveries of the 20th century was antibiotics, which allowed the extension of human life facing severe illness and, often, premature death. Unfortunately, the onset of resistance phenomena to antibiotics is a natural consequence of the evolution of bacteria, mostly due to their widespread use/misuse not only in humans but also in animals and crops. Additionally, cancer is a disease that has not yet been defeated. The increasing recurrence of tumors, severe side-effects, and resistance phenomena represent the major issues impacting the clinical efficacy of most of the anticancer agents currently in use. The most important current challenge is to find multitarget agents acting simultaneously against different pathways of cancer and microbial infection, and Ag(I)–NHC complexes offer a valid cue for developing new classes of drugs. In this scenario, Ag(I)–NHC complexes received particular attention due to their interesting biological potential since several compounds belonging to this class have shown antimicrobial or anticancer potential or both. This review summarizes the most recent studies in this view, regarding complex **SBC3** and some silver(I)–NHC complexes. Interesting results have been obtained for Ag(I)–NHC complexes as antimicrobials against both fungi (*C. albicans*, *C. parapsilosis,* and *C. glabrata*) and bacteria (Gram-positive: *S. aureus*, MRSA, *E. faecalis*, *S. epidermidis*, *M. luteus*, *and B. subtilis;* and Gram-negative: *P. aeruginosa*, *E. coli*, *S. typhimurium*, *K. pneumoniae,* and *S. typhi*), as well as against clinical isolates and anticancers against breast, colorectal, lung, prostate, cervical cancer, leukemia, and neuroblastoma cell lines. The synthesis involved silver oxide or silver acetate in dichloromethane, methanol, or a mixture of toluene/methanol, leading to complexes in moderate to high yields (the highest yields were ≥87%). The mechanism of action for these compounds has not yet been definitively elucidated, although various hypotheses have been suggested. For the antimicrobial activity, it seems that higher lipophilicity is required and that the complexes should retain their ligands by releasing silver cations for a prolonged time. For **SBC3**, the increase in the abundance of chitinase was also suggested as being involved in the mechanism of action of this compound as antifungal against *C. parapsilosis*, whereas in *P. aeruginosa*, a multitude of pathways were affected, including alginate biosynthesis, secretion systems, drug detoxification, and anaerobic respiration. This was in contrast to the response of *S. aureus*, where pathways such as protein synthesis, glucose metabolism, and cell redox homeostasis were affected. The anticancer activity of silver(I)–NHC complexes is likely related to the inhibition of the selenoenzyme thioredoxin reductase (TrxR) or the ability to interfere with critical cellular processes, including apoptosis activation or DNA replication and repair. Some recent suggestions have been carried out regarding a potential interaction with aromatase cytochrome P450, the inhibition of topoisomerases I and II, and interference with the cytoskeleton dynamic and a reduction in TNF-α expression, which has been found to be highly expressed in different preneoplastic and tumor tissues. Thus, more studies are needed to further comprehend the mechanism of action of these drugs. Moreover, generally, to date, only a few studies have addressed the discovery of compounds behaving both as antimicrobials and anticancers. Few in vivo studies and no clinical studies on these compounds have been conducted so far. The overall conclusion drawn from this review is that the NHC core confirms its relevance for biological activities, as well as silver. Moreover, researchers and pharmaceutical companies are required to work together, aiming at the discovery and utilization of new and effective compounds, such as Ag(I)–NHC complexes, acting together against diverse types of cancer and multi-resistant bacteria.

## Figures and Tables

**Table 2:** Ag–NHC complexes as anticancer agents.

Structure	Compd	Anticancer Activity	Ref.
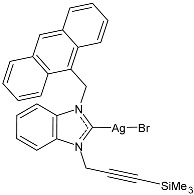	**17**	IC_50_ = 7 ± 2 µM (SW480) IC_50_ = 10 ± 1 µM (A549) IC_50_ = 8 ± 1 µM (HepG2)	[79]
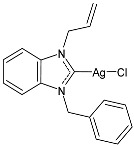	**18**	IC_50_ = <1 µM (MCF-7) IC_50_ = 2.74 ± 0.32 µM (DU-145) IC_50_ = <1 µM (MDA-MB-231)	[80]
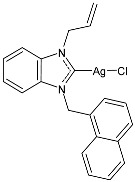	**19**	IC_50_ = <1 µM (MCF-7) IC_50_ = 2.11 ± 0.05 µM (DU-145) IC_50_ = 1.26 ± 0.02 µM (MDA-MB-231)	[80]
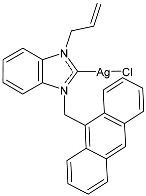	**20**	IC_50_ = 1.02 ± 0.05 µM (MCF-7) IC_50_ = 1.74 ± 0.21 µM (DU-145) IC_50_ = <1 µM (MDA-MB-231)	[80]
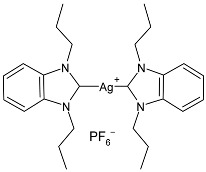	**21**	IC_50_ = 0.31 µM (HCT-116) IC_50_ = 15.1 µM (MCF-7) IC_50_ = 7.0 µM (K-562)	[81]
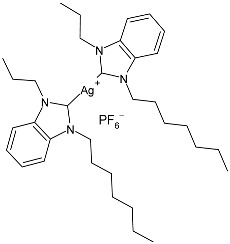	**22**	IC_50_ = 15.1 µM (HCT-116) IC_50_ = 16.1 µM (MCF-7) IC_50_ = 17.9 µM (K-562)	[81]
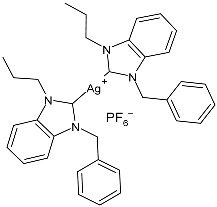	**23**	IC_50_ = 1.99 µM (HCT-116) IC_50_ = 35.2 µM (MCF-7) IC_50_ = 10.7 µM (K-562)	[81]
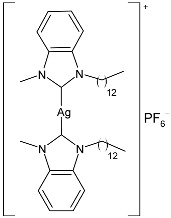	**24**	IC_50_ = 1.04 ± 0.21 µg/mL; 1.22 µM (HeLa)	[82]
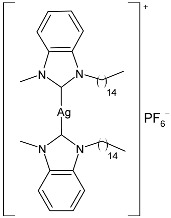	**25**	IC_50_ = 1.07 ± 0.08 µg/mL; 1.18 µM (HeLa)	[82]
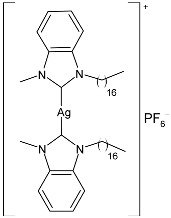	**26**	IC_50_ = 1.14 ± 0.18 µg/mL; 1.18 µM (HeLa)	[82]
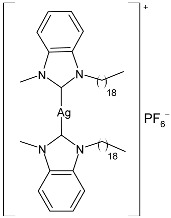	**27**	IC_50_ = 2.27 ± 0.04 µg/mL; 2.22 µM (HeLa)	[82]
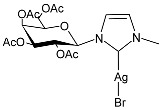	**28**	IC_50_ = 34.5 µM (RMS cell line, RD)	[83]
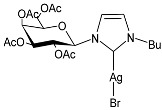	**29**	IC_50_ = 28.2 µM (RMS cell line, RD)	[83]
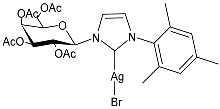	**30**	IC_50_ = 26.7 µM (RMS cell line, RD)	[83]
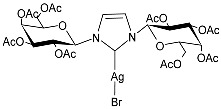	**31**	IC_50_ = 20.4 µM (RMS cell line, RD)	[83]
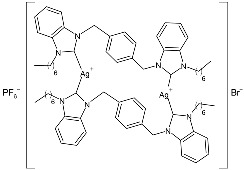	**32**	IC_50_ = 1.087 ± 0.05 µg/mL (MCF-7) IC_50_ = 0.986 ± 0.05 µg/mL (A549) IC_50_ = 0.962 ± 0.08 µg/mL (A-2780) IC_50_ = 1.023 ± 0.07 µg/mL (HCT-116)	[84]
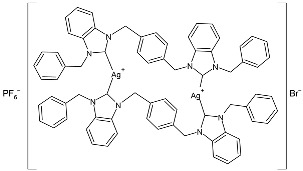	**33**	IC_50_ = 0.996 ± 0.04 µg/mL (MCF-7) IC_50_ = 0.975 ± 0.04 µg/mL (A549) IC_50_ = 0.872 ± 0.05 µg/mL (A-2780) IC_50_ = 0.987 ± 0.05 µg/mL (HCT-116)	[84]
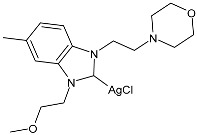	**34**	IC_50_ = 6.26 ± 0.30 µM (HepG2) IC_50_ = 3.25 ± 0.12 µM (A549) IC_50_ = 5.74 ± 0.12 µM (MCF-7)	[85]
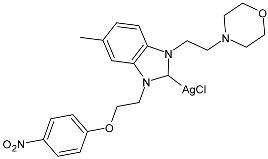	**35**	IC_50_ = 6.21 ± 0.08 µM (HepG2) IC_50_ = 1.65 ± 0.05 µM (A549) IC_50_ = 5.46 ± 0.16 µM (MCF-7)	[85]
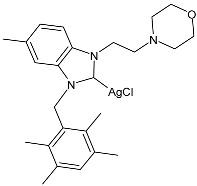	**36**	IC_50_ = 12.62 ± 0.55 µM (HepG2) IC_50_ = 6.49 ± 0.06 µM (A549) IC_50_ = 8.43 ± 0.27 µM (MCF-7)	[85]
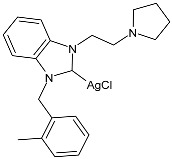	**37**	IC_50_ = 5.37 ± 0.20 µM (HepG2) IC_50_ = 3.34 ± 0.13 µM (A549) IC_50_ = 5.83 ± 0.11 µM (MCF-7)	[86]
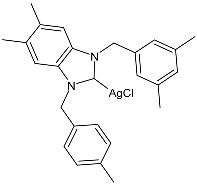	**38**	IC_50_ = 4.44 ± 0.07 µM (HepG2) IC_50_ = 1.77 ± 0.04 µM (A549) IC_50_ = 4.58 ± 0.14 µM (MCF-7)	[86]
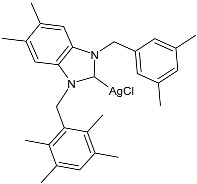	**39**	IC_50_ = 11.82 ± 0.59 µM (HepG2) IC_50_ = 6.09 ± 0.08 µM (A549) IC_50_ = 9.54 ± 0.28 µM (MCF-7)	[86]

IC_50_ = half-maximal (50%) inhibitory concentration.

**Table 3:** Ag–NHC as dual antibacterial and anticancer agents.

Structure	Compd	Antibacterial Activity	Anticancer Activity	Ref.
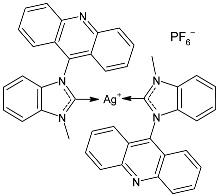	**40**	MIC = 250 mg/mL (*S. aureus* ATCC 25923) MIC = 250 mg/mL (*S. epidermidis* clinical isolate) MIC = 250 mg/mL (*P. aeruginosa* ATCC27853) MIC = 250 mg/mL (*E. coli* clinical isolate) MIC = 250 mg/mL (*S. typhi* clinical isolate)	IC_50_ = 20 ± 3 mg/mL; 22 µM (MCF-7)	[87]
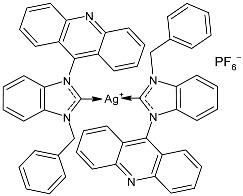	**41**	MIC = 250 mg/mL (*S. aureus* ATCC 25923 MIC = 250 mg/mL (*S. epidermidis* clinical isolate) MIC = 250 mg/mL (*P. aeruginosa* ATCC27853) MIC = 250 mg/mL (*E. coli* clinical isolate) MIC = 250 mg/mL (*S. typhi* clinical isolate)	IC_50_ = 22 ± 3 mg/mL; 21 µM (MCF-7)	[87]
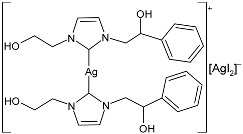	**42**	MIC = 15 µg/mL; 32.1 µM (*E. coli*) MIC = 50 µg/mL; 107.0 µM (*S. aureus*)	IC_50_ > 200 µM (MDA-MB-231) IC_50_ = 20.3 ± 1.1 µM (MCF-7) IC_50_ = 12.2 ± 1.0 µM (HeLa)	[53]
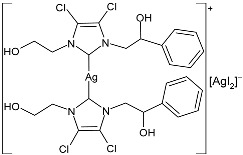	**43**	MIC = 15 µg/mL; 27.9 µM (*E. coli*) MIC > 150 µg/mL; > 279.8 µM (*S. aureus*)	IC_50_ > 200 µM (MDA-MB-231) IC_50_ = 19.5 ± 0.9 µM (MCF-7) IC_50_ 11.9 ± 0.4 µM (HeLa)	[53]
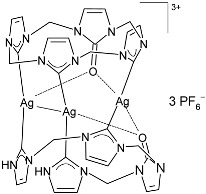	**44**	MIC = 30 µM (*S. aureus*) MIC = 25–50 µM (*E. coli*)	IC_50_ = 3.61 ± 1.04 µM (HeLa) IC_50_ = 3.03 ± 1.06 µM (MCF-7)	[88]
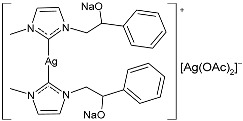	**45**	MIC = 5 µg/mL (*E. coli*) MIC = 5 µg/mL (*S. aureus*) MIC = 10 µg/mL (*E. faecalis*)	IC_50_ = 7.0 ± 0.4 µM (MDA-MB-231) IC_50_ = 18.3 ± 0.8 µM (MCF-7)	[89]
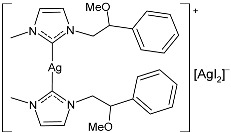	**46**	MIC = 5 µg/mL (*E. coli*) MIC = 5 µg/mL (*S. aureus*) MIC = 10 µg/mL (*E. faecalis*)	IC_50_ = 52.6 ± 0.3 µM (MDA-MB-231) IC_50_ = 31.8 ± 0.8 µM (MCF-7)	[89]
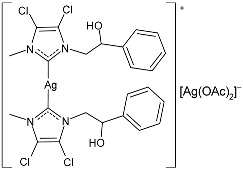	**47**	MIC = 10 µg/mL (*E. coli*) MIC = 5 µg/mL (*S. aureus*) MIC = 10 µg/mL (*E. faecalis*)	IC_50_ = 38.1 ± 0.8 µM (MDA-MB-231) IC_50_ = 13.2 ± 0.3 µM (MCF-7)	[89]
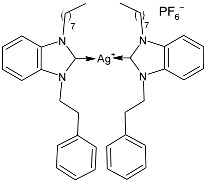	**48**	MIC = 0.15 ± 0.03 µg/mL (*S. aureus*) MIC = 0.073 ± 0.02 µg/mL (*M. luteus*) MIC = 0.12 ± 0.05 µg/mL (*E. coli*) MIC = 0.095 ± 0.02 µg/mL (*S. typhimurium*)	IC_50_ = 1.1691 ± 0.12 µM (MCF-7) IC_50_ = 1.116 ± 0.09 µM (HCT-116) IC_50_ = 0.9850 ± 0.08 µM (A549)	[90]
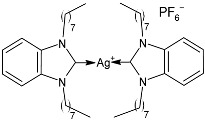	**49**	MIC = 0.035 ± 0.01 µg/mL (*S. aureus*) MIC = 0.11 ± 0.05 µg/mL (*M. luteus*) MIC = 0.21 ± 0.03 µg/mL (*E. coli*) MIC = 0.15 ± 0.06 µg/mL (*S. typhimurium*)	IC_50_ = 0.9813 ± 0.09 µM (MCF-7) IC_50_ = 1.101 ± 0.14 µM (HCT-116) IC_50_ = 0.9730 ± 0.12 µM (A549)	[90]
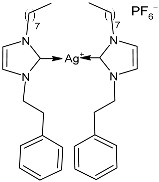	**50**	MIC = 0.046 ± 0.01 µg/mL (*S. aureus*) MIC = 0.035 ± 0.04 µg/mL (*M. luteus*) MIC = 0.13 ± 0.08 µg/mL (*E. coli*) MIC = 0.054 ± 0.01 µg/mL (*S. typhimurium*)	IC_50_ = 1.3787 ± 0.17 µM (MCF-7) IC_50_ = 1.299 ± 0.13 µM (HCT-116) IC_50_ = 1.1773 ± 0.17 µM (A549)	[90]
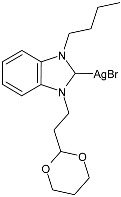	**51**	IZD = not active (*B. subtilis*) IZD = not active (*E. coli*)	IC_50_ = 6.01 ± 2.39 µg/mL (MCF-7)	[91]
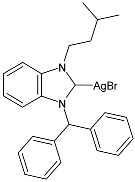	**52**	IZD = 7.33 ± 0.57 mm (*B. subtilis*) IZD = 9.33 ± 1.15 mm (*E. coli*)	IC_50_ = 11.58 ± 2.58 µg/mL (MCF-7)	[91]
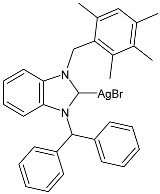	**53**	IZD = 10.33 ± 1.52 mm (*B. subtilis*) IZD = 16.00 ± 2.00 mm (*E. coli*)	IC_50_ = 3.40 ± 0.87 µg/mL (MCF-7)	[91]
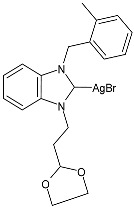	**54**	IZD = 5.33 ± 1.15 mm (*B. subtilis*) IZD = 8.67 ± 1.15 mm (*E. coli*)	IC_50_ = 9.08 ± 2.91 µg/mL (MCF-7)	[91]
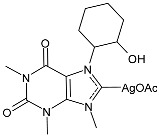	**55**	MIC = 100 µg/mL (*S. aureus*) MIC = 150 µg/mL (*E. faecalis)* MIC = 100 µg/mL (*S. epidermidis)* MIC = 100 µg/mL (*E. coli*) MIC = 125 µg/mL (*K. pneumoniae*) MIC = 100 µg/mL (*P. aeruginosa*) MIC = 150 µg/mL (*S. typhimurium*)	IC_50_ = 19.4 ± 1 µM (MDA-MB-231) IC_50_ = 28.7 ± 1 µM (MCF-7)	[92]
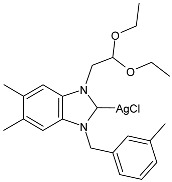	**56**	MIC = 12.5 µg/mL (*C. albicans*) MIC = 6.25 µg/mL (*C. glabrata*) MIC = 25 µg/mL (*E. coli*) MIC = 25 µg/mL (*P. aeruginosa*) MIC = 35 µg/mL (*S. aureus*)	IC_50_ = 35.82 ± 0.43 µM (A549) IC_50_ = 40.29 ± 1.26 µM (MCF-7) IC_50_ = 32.58 ± 0.63 µM (HCT-116) IC_50_ = 47.75 ± 0.63 µM (SH-SY5Y)	[93]

MIC = minimum inhibitory concentration; IZD = inhibitory zone diameter; IC_50_ = half-maximal (50%) inhibitory concentration.

## Abbreviations and Cell Lines

A-2780, ovarian cancer cells
A549, lung cancer cells
*A. baumanii* = *Acinetobacter baumanii*
BEAS-2B, bronchial epithelial cells
*C. albicans* = *Candida albicans*
*C. parapsilosis* = *Candida parapsilosis*
DU-145, prostate cancer cells
*E. faecium* = *Enterococcus faecium*
HCT-116, human colorectal cancer cells
Hek-293, normal embryonic kidney cells
HeLa, human cervical cancer cells
Hs27, normal human skin fibroblasts
IC_50_ = half-maximal (50%) inhibitory concentration
IMR-90, lung fibroblasts
IZD = inhibitory zone diameter
*K. pneumoniae* = *Klebsiella pneumoniae*
K-562, erythromyeloblastoid leukemia cells
*M. bovis* = *Mycobacterium bovis*
MDA-MB-231, breast cancer cells
MGI = Maximum growth inhibition
MIC = minimum inhibitory concentration
MRSA = methicillin-resistant *S. aureus*
MSSA *=* methicillin-sensitive *S. aureus*
*M. smegmatis* = *Mycobacterium smegmatis*
MCF-7, breast cancer cells
MCF-10A, normal breast cells
MTT = 3-(4,5-dimethylthiazol-2-yl)-2,5-diphenyltetrazolium bromide)
NHC = *N*-heterocyclic carbene
*P. aeruginosa* = *Pseudomonas aeruginosa*
RMS, rhabdomyosarcoma cells
*S. aureus* = *Staphylococcus aureus*
SDS = sodium dodecyl sulfate
*S. epidermidis* = *Staphylococcus epidermidis*
SH-SY5Y, neuroblastoma cancer cells
*S. typhi* = *Salmonella typhi*
*S. typhimurium* = *Salmonella typhimurium*
SW480, colon cells
ZC = zone of clearance
